# Sarcopenia and osteo‐sarcopenia: Nonnegotiable patient‐related aspects to consider when comparing kinematic and mechanical alignment strategies for total knee arthroplasty

**DOI:** 10.1002/jeo2.70304

**Published:** 2025-06-08

**Authors:** Francesco Pegreffi, Maria Tiziana di Leo, Gianluca Zocco, Gianluca Giuseppe Costa, Arcangelo Russo, Raoul Saggini

**Affiliations:** ^1^ Department of Medicine and Surgery University of Enna Kore Enna Italy; ^2^ Recovery and Functional Rehabilitation Unit Umberto I Hospital Enna Italy; ^3^ Orthopaedic and Traumatology Unit Umberto I Hospital Enna Italy; ^4^ Faculty of Psychology 471917 eCampus University Novedrate Italy

**Keywords:** alignment strategies, osteo‐sarcopenia, rehabilitation, sarcopenia, total knee arthroplasty

**Abbreviations**
BMD: bone mineral density
OA: osteoarthritis
TKA: total knee arthroplasty

Dear Editor,

We read with great interest the recent article by Jamali et al., titled ‘Kinematic Versus Mechanical Alignment: A Systematic Review of Systematic Reviews and Meta‐Analyses of Randomised Controlled Trials’ [2].

The authors provide a comprehensive and well‐structured analysis of the strengths and limitations of kinematic versus mechanical alignment strategies in total knee arthroplasty (TKA), raising crucial concerns.

Their work underscores how the evaluation of these two alignment surgical techniques remains challenging, highlighting the absence of a standardised methodology for patient assessment, which hinders the accurate identification of biases and limits the ability to properly scrutinise patient populations [2].

We fully align with the conclusions of this elucidating study. However, we believe that an equally crucial yet often overlooked factor in assessing TKA success is the muscular status of the patient, which remains underestimated despite its significant impact on functional outcomes and recovery.

The evaluation of muscle mass and function should be considered as important as surgical technique, as both directly influence postoperative rehabilitation and functional recovery. Sarcopenia and osteo‐sarcopenia, characterised by progressive muscle mass loss and decreased bone density, have been increasingly recognised as critical determinants of mobility, balance, and post‐surgical recovery [2].

Several studies have identified sarcopenia as a key contributor to poor functional outcomes following TKA, independent of surgical alignment technique [5]. Recent evidence further supports this connection, showing that muscle mass gradually increases after TKA, with a subsequent delayed increase in bone mineral density (BMD) occurring after 12 months postoperatively [1]. A prospective study of patients with end‐stage knee osteoarthritis found a significant positive correlation between the increase in muscle mass and the increase in BMD, suggesting that postoperative muscle recovery may play a pivotal role in bone health restoration after TKA [1]. These findings highlight the dynamic interplay between muscle and bone health in TKA patients, reinforcing the need to incorporate muscular status as a fundamental consideration in evaluating surgical outcomes.

While alignment strategies such as kinematic or mechanical approaches undoubtedly play a crucial role in knee function restoration, failing to account for the musculoskeletal health of the patient, particularly sarcopenia, may lead to suboptimal rehabilitation and increased risk of complications. Recent evidence suggests that the prevalence of sarcopenia in knee osteoarthritis (OA) patients is significantly higher than in non‐OA populations, affecting nearly 45.2% of patients compared to 31.2% in controls [4]. A systematic review and meta‐analysis demonstrated that sarcopenia in knee OA is more than twice as prevalent as in healthy individuals (odds ratio = 2.07, 95% confidence interval: 1.43–3.00) [4]. This heightened prevalence underscores the need for greater attention to muscle health in preoperative assessment and postoperative rehabilitation strategies for TKA patients.

Despite its clinical importance, sarcopenia assessment remains challenging due to the complexity and cost of diagnostic tools. While magnetic resonance imaging is one of the most accurate methods for evaluating muscle quality, it is rarely utilised in routine orthopaedic assessments due to its high cost and limited availability [3]. Other imaging modalities, such as dual‐energy X‐ray absorptiometry and computed tomography, involve radiation exposure and are not standard in orthopaedic workflows. Nonradiation techniques, such as ultrasound and bioelectrical impedance analysis, offer promising alternatives, but they require standardised protocols to ensure reliability [3].

Given these challenges, sarcopenia screening is often overlooked in TKA candidates, potentially leading to unrecognised risk factors for poor functional recovery.

The study by Jamali et al. highlights the need for scrutinising systematic reviews and meta‐analyses on kinematic and mechanical alignment strategies in TKA. The authors correctly conclude that the current literature is inadequate to determine if kinematc alignment offers a true advantage over mechanic, and that claims in systematic reviews should be carefully assessed based not only on bias risks but also on study populations and surgical methodology [5].

While this is essential, we believe that an equally critical factor remains overlooked the muscular status of the patient.

The assessment of muscle function and sarcopenia should be considered as important as surgical technique when evaluating the success of different alignment approaches. Postoperative function is influenced not only by the precision of surgical alignment but also by the patient's baseline muscle quality and strength, which directly affect rehabilitation potential and long‐term outcomes.

I commend the authors for tackling this challenging and still underexplored topic and encourage further interdisciplinary research that integrates orthopaedic biomechanics with musculoskeletal health assessments.

Future studies should aim to develop standardised preoperative sarcopenia screening protocols and investigate the impact of muscle quality on TKA outcomes, ensuring a more holistic approach to knee arthroplasty success.

## AUTHOR CONTRIBUTIONS

All authors contributed equally to the preparation and review of this letter.

## CONFLICT OF INTEREST STATEMENT

The authors declare no conflicts of interest.
